# Rheology Modifying Reagents for Clay-Rich Mineral Suspensions: A Review

**DOI:** 10.3390/polym17172427

**Published:** 2025-09-08

**Authors:** Williams Leiva, Norman Toro, Pedro Robles, Gonzalo R. Quezada, Iván Salazar, Javier Flores-Badillo, Ricardo I. Jeldres

**Affiliations:** 1 Facultad de Ingeniería, Universidad San Sebastián, Sede Concepción, Concepción 4030000, Chile; 2 Faculty of Engineering and Architecture, Universidad Arturo Prat, Iquique 1100000, Chile; notoro@unap.cl; 3 Escuela de Ingeniería Química, Pontificia Universidad Católica de Valparaíso, Valparaíso 2340000, Chile; 4 Departamento de Ingeniería en Maderas, Facultad de Ingeniería, Universidad del Bío-Bío, Concepción 4030000, Chile; grquezada@ubiobio.cl; 5 Departamento de Ingeniería Civil, Universidad Católica del Norte, Antofagasta 1270709, Chile; isalazar@ucn.cl; 6 Área Académica de Ciencias de la Tierra, Universidad Autónoma del Estado de Hidalgo, Carretera Pachuca-Tulancingo km. 4.5, Colonia Carboneras, Mineral de la Reforma C.P. 42184, Hidalgo, Mexico; javier_flores11060@uaeh.edu.mx; 7 Departamento de Ingeniería Química y Procesos de Minerales, Facultad de Ingeniería, Universidad de Antofagasta, Antofagasta 1240000, Chile; ricardo.jeldres@uantof.cl; 8 Advanced Mininig Technology Center (AMTC), Universidad de Antofagasta, Antofagasta 1240000, Chile

**Keywords:** clay suspensions, surfactants, polymers, nanoparticles, rheology

## Abstract

In the mining industry, key unit operations such as grinding, flotation, thickening, and tailings transport are negatively affected by the presence of clay minerals, which impart complex rheological behaviors to mineral suspensions by increasing their rheological properties. This deterioration arises from specific physicochemical characteristics of clay minerals such as fine particle size, anisotropic character, laminar morphology, and swelling capacity. This work reviews the effects of various rheology-modifying reagents on clay suspensions including kaolinite, illite, and montmorillonite. The reviewed reagents include inorganic salts, pH modifiers, polymers, surfactants, and nanoparticles. Their mechanisms of interaction with solid particles are analyzed, highlighting their influence on the degree of dispersion or aggregation. Furthermore, this review proposes research opportunities focused on the formulation of hybrid reagents, modified biopolymers, and the development of reagents effective under adverse conditions such as high salinity or elevated temperatures. This review provides a comprehensive basis for optimizing the use of rheological additives through more efficient and sustainable strategies for managing clay-rich suspensions in the mining industry.

## 1. Introduction

The mining industry processes large volumes of slurries that contain a significant proportion of fine particles. This is mainly due to the need for finer grinding resulting from declining ore grades. Fine particles interact with each other and with the chemical conditions of the aqueous phase, leading to either aggregation or dispersion, which affects their rheological behavior. Additionally, ores often contain clay minerals, which further exacerbate changes in the pulp’s rheological properties. Consequently, the complex rheological behavior of mineral pulps can negatively impact several mineral processing operations such as grinding, flotation, thickening, and tailings transport.

In grinding, rheology influences both the production rate of fine material and the classification efficiency of hydrocyclones [1]. Shi and Napier-Munn [2] showed that pulp rheology affects industrial grinding by influencing the grinding index depending on whether the pulp behaves as a dilatant or pseudoplastic fluid, and whether it exhibits high or low yield stress. In stirred mills, yield stress is the dominant rheological parameter; as it increases, energy consumption rises, breakage rates decrease, fine production declines, and the particle size distribution becomes narrower [3]. Several studies have demonstrated that pulp rheology impacts wet milling performance [4,5,6,7,8,9], especially in tumbling ball mills [2,10,11,12,13,14] and has garnered increasing interest in ultrafine grinding performance [15,16,17,18,19].

In flotation, solid concentration alters pulp rheology and thereby impacts the hydrodynamics of flotation cells [20]. Higher solid concentrations lead to reduced bubble size and gas retention due to the formation of a “pulp cavern” around the impeller (caused by yield stress), which generates smaller bubbles with poor dispersion [21]. Increased pulp viscosity also hinders aeration and the upward motion of mineralized bubbles to the froth layer [22]. Low apparent viscosity decreases turbulence damping and increases local energy dissipation near the impeller stream, a key parameter that directly influences aggregate stability and shear forces acting on particle–bubble aggregates [23,24]. In general, high pulp viscosity leads to poor bubble dispersion, reduced particle–bubble collision efficiency, and increased turbulence damping, negatively impacting valuable mineral recovery [21,25,26].

In thickening and tailings transport, the management of thickened tailings is constrained by pulp rheology, which affects thickener underflow discharge, pumping energy costs, and deposition in tailings storage facilities (TSFs). Thickened tailings have high solids content but also present complex fluid behavior and significant friction losses (hydraulic gradient) compared to conventional tailings. These tailings behave as Bingham-type non-Newtonian fluids, characterized by a yield stress. While high yield stress enables laminar flow over long distances, avoiding particle settling in pipelines [27,28]—it also requires higher pumping pressures, raising capital and operational costs. Excessive yield stress may cause poor tailings distribution due to steep repose angles [29], although it enhances physical stability post-deposition. Rudman et al. [30] reported a near-linear relationship between thickener rake torque and yield stress over a range of rake speeds. Excessive torque may cause operational issues, including rake stall and thickener bed buildup [31].

Clay minerals in gangue materials introduce widespread adverse effects across various stages of mineral processing. These minerals possess non-spherical particle shapes and complex rheological behaviors, unlike spherical particles [32]. The issues are both physicochemical and pervasive throughout the mineral processing circuit, affecting slurry transport, beneficiation, water recovery, and tailings disposal [33]. Phyllosilicates consist of tetrahedral (T) and octahedral (O) layers, whose stacking and interlayer bonding define their classification [34]. They are grouped into serpentines, talc/pyrophyllite, micas, chlorites, and clay minerals. Among them, clay minerals are of particular interest due to their anisotropy, colloidal size, and unique features such as high aspect ratio, surface heterogeneity, swelling potential, cation exchange capacity, and layer delamination [35], all of which contribute to complex rheological behaviors.

Due to their anisotropic nature, clay minerals exhibit poor settling and stacking, often forming low-density “house-of-cards” aggregates [36,37,38]. Rheological complexities in swelling clays like bentonite and montmorillonite arise from: (i) house-of-cards network formation [38,39] and (ii) swelling behavior [40,41]. Swelling is caused by highly hydrated interlayer cations (e.g., ${\mathrm{Na}}^{+}$, ${\mathrm{Li}}^{+}$), which attract water molecules and force apart the TOT layers [40,41,42]. This leads to an effective increase in suspension volume fraction and enhanced particle–particle interactions, which drastically impact the rheology.

The need to modify the rheological properties of mineral slurries presents an opportunity to enhance the operation and performance of mineral processing operations, which can be achieved through the addition of chemical additives. These additives can be either organic or inorganic and interact with particle surfaces, affecting their state of aggregation or dispersion. Organic rheology modifiers, including polymers and surfactants, act through a combination of electrostatic and steric forces. A low-molecular-weight polymer functions as a dispersant by providing both electrostatic and steric repulsion. The latter involves the adsorption of the polymer onto particle surfaces, creating a physical barrier that prevents particles from approaching distances at which van der Waals attractive forces can act. This results in a reduction in both the yield stress and apparent viscosity of the suspension. Medium- and high-molecular-weight polymers act as flocculants, with the former promoting charge neutralization (coagulation) and the latter facilitating the formation of interparticle bridges.

In contrast, inorganic modifiers alter the surface charge of mineral particles, thereby controlling the magnitude of electrostatic attraction or repulsion between them. These interactions can be either physical or chemical: physical interactions arise from electrostatic forces, whereas chemical interactions occur due to the high affinity of certain ions for the mineral surface. For instance, a pH modifier may behave as either a dispersant or a coagulant depending on its dosage. At a pH near the isoelectric point, it may induce coagulation, whereas at a pH far from this point, it may promote electrostatic repulsion, leading to dispersion.

Mineral suspensions with high clay content exhibit complex rheological behavior, posing significant operational challenges in mineral processing. This complexity is reflected in increased viscosity, the formation of gel-like structures, and elevated yield stresses, all of which directly affect the performance of unit operations such as flotation (due to impaired bubble dispersion and particle–bubble collisions), thickening (due to reduced settling rates and increased thickener rake torque), and tailings transport (due to greater head losses and intensified pumping requirements). These issues can potentially be mitigated through the addition of rheological modifiers such as polymers, surfactants, salts, and nanoparticles.

Numerous studies have investigated the effects of rheological additives on clay-rich suspensions; however, there remains a lack of critical and comparative analyses that systematically elucidate their mechanisms of action, relative effectiveness, and limitations under emerging industrial conditions, such as high salinity (linked to seawater use amid freshwater scarcity), finer particle sizes, and pH variations. In this context, the central question guiding this review is: Which types of rheological additives have proven effective in modifying the behavior of clay suspensions in mining applications, under which physicochemical conditions, and what knowledge gaps persist regarding their understanding and practical implementation?. This work aims to address that question through an analytical review of the main reagents used to modify the rheology of clay suspensions, with a focus on their interactions with minerals such as kaolinite, illite, and montmorillonite. The review identifies both the potential operational improvements that these additives may offer and their current limitations, ultimately proposing future research directions aimed at developing more efficient, sustainable, and adaptable additives for complex operational scenarios.

### Review Strategy

This review was conducted through a systematic search of scientific databases, including Scopus, Web of Science, and ScienceDirect, focusing on publications, conference proceedings, and books from 1999 to 2025. Keyword combinations used in the search included terms such as “clay rheology,” “rheology modifiers,” “polymers + clay suspensions,” “surfactants + bentonite,” and “nanoparticles + tailings,” among others.

The review focused on research articles presenting experimental data related to the rheological behavior of suspensions containing clay minerals, mainly within the context of mineral processing. However, studies from other industries where clay manipulation is critical for rheological control were also considered, including:

(i) the oil and gas industry, particularly in the design of bentonite-based drilling fluids;
(ii) the ceramics industry, where flow properties of suspensions containing kaolinite and other clays are regulated;
(iii) the chemical and materials industries, involving the development of nanocomposites, functional surfactants, and hybrid additives; and
(iv) environmental and wastewater treatment, where rheological properties influence the efficiency of sedimentation and clarification processes.

The review excluded theoretical studies without experimental validation, publications exclusively related to sectors unrelated to mineral suspensions, and reports without full-text access. In total, over 200 bibliographic sources were reviewed, of which 157 were selected based on their relevance, originality, and applicability to the context of mineral processing involving clay-bearing systems.

## 2. General Principles

### 2.1. Clay Minerals

**Figure 1:**
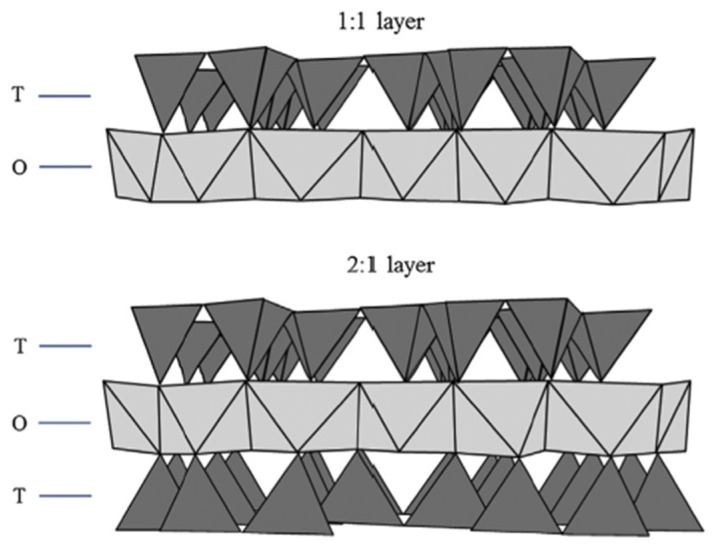
Schematic representation of the structure of a clay mineral. T and O correspond to the tetrahedral and octahedral layers, respectively.

**Figure 2:**
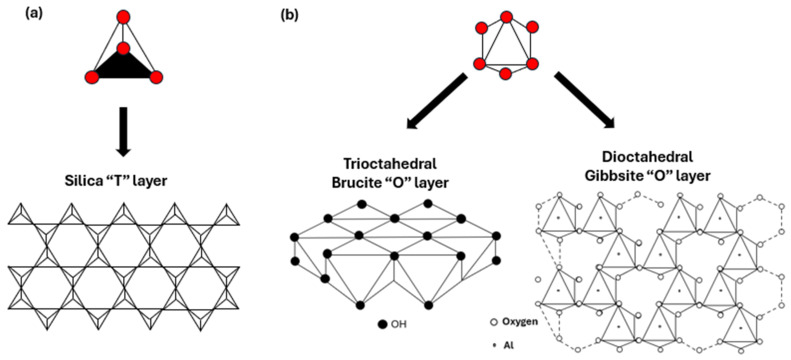
(**a**) $\mathrm{Si}O_{4}$ tetrahedral units and silica ‘T’ layers (**b**) octahedral ($XO_{6}$) units and the difference between brucite and gibbsite ‘O’ layers. Adapted from [33].

Clay minerals refer to phyllosilicate minerals and related materials that impart plasticity to clays and harden upon drying or firing [43]. Their structure consists of tetrahedral (T) and octahedral (O) sheets, which combine in a 1:1 or 2:1 ratio (Figure 1), resulting in TO or TOT anisotropic layers. These layers may carry negative charges (as in most clay minerals), positive charges (as in layered double hydroxides), or be neutral (as in talc and pyrophyllite). Each tetrahedron comprises a cation coordinated to four oxygen atoms, and these units share three basal oxygen atoms with adjacent tetrahedra, forming an infinite two-dimensional hexagonal mesh (Figure 2a). In the octahedral sheet, edge-sharing between adjacent octahedra produces sheets with hexagonal or pseudo-hexagonal symmetry (Figure 2b).

In the 1:1 structure, the tetrahedral sheet consists mainly of silica $({\left({\mathrm{Si}}_{2}O_{5}\right)}^{2-}),$ while the octahedral sheet typically comprises alumina (${\left[{\mathrm{Al}}_{2}{\left(\mathrm{OH}\right)}_{4}\right]}^{2+})$. The apical oxygens of the silica layer are shared with aluminum atoms in the octahedral layer below. In 2:1 structures, the octahedral layer is sandwiched between two tetrahedral layers (Figure 1). Octahedral sheets may be either trioctahedral (e.g., brucite, $\mathrm{Mg}{\left(\mathrm{OH}\right)}_{2}$) when occupied by divalent cations, or dioctahedral (e.g., gibbsite, $\mathrm{Al}{\left(\mathrm{OH}\right)}_{3}$) when trivalent cations dominate (Figure 2).

When these structures are broken, two types of surfaces are exposed: (i) a basal face resulting from cleavage between stacked layers, and (ii) an edge surface due to rupture of ionic or covalent bonds within the layer. The basal surfaces possess permanent negative charges, largely due to isomorphic substitution of higher valence ions with lower valence ions (e.g., Si^4+^→Al^3+^). Edge surfaces tend to be positively charged at acidic to neutral pH levels, depending on the clay mineral type [37].

The morphological nature of clay minerals leads to three primary aggregation modes: (i) Face-to-face (FF) which produces layered structures with low specific volume and low yield stress, (ii) Edge-to-face (EF), and (iii) Edge-to-edge (EE) which result in three-dimensional “house-of-cards” structures with higher apparent volume fractions [44,45].

According to Deer et al. [34], clay minerals can be categorized into non-swelling clays (kaolinites and illites) and swelling clays (smectites and vermiculites). Dixon and Weed [46] also include chlorites in the clay group. Kaolinite, illite, and smectite are the most encountered clay minerals in association with valuable ores such as copper and gold [47]. Kaolinite belongs to the kaolin group and has a 1:1 dioctahedral structure with a general composition of ${\mathrm{Al}}_{2}{\mathrm{Si}}_{2}O_{5}{\left(\mathrm{OH}\right)}_{4}$. It is characterized by predominant ${\mathrm{Al}}^{3+}$ in octahedral sites, although isomorphic substitution by ${\mathrm{Fe}}^{3+}$, ${\mathrm{Mg}}^{2+}$, or ${\mathrm{Ti}}^{4+}$ can occur. Illite is commonly associated with sedimentary rocks and is recognized as a non-expandable 2:1 mica-smectite-type mineral. The term “illite” refers to a series of compositions; Rieder et al. [48] recommend its use as a mineral series name. Its structural unit is similar to muscovite, with an “O” gibbsite-like layer flanked by inward-facing “T” silica layers (T-O-T configuration). Smectites are 2:1 phyllosilicates with a total negative layer charge of 0.2–0.6 per half unit cell. The octahedral sheet may be occupied predominantly by trivalent cations (dioctahedral smectites) or divalent cations (trioctahedral smectites). Tetrahedral, octahedral, and interlayer sites can accommodate a broad range of cations. Commonly, ${\mathrm{Si}}^{4+}$, ${\mathrm{Al}}^{3+}$, and ${\mathrm{Fe}}^{3+}$ are found on tetrahedral sites while ${\mathrm{Al}}^{3+}$, ${\mathrm{Fe}}^{3+}$, ${\mathrm{Fe}}^{2+}$, ${\mathrm{Mg}}^{2+}$, ${\mathrm{Ni}}^{2+}$, ${\mathrm{Zn}}^{2+}$ and ${\mathrm{Li}}^{+}$ generally occupy octahedral sites. In dioctahedral smectites, substitution of divalent by trivalent cations generates excess negative charge, whereas in trioctahedral smectites, the reverse substitution can induce positive charge. These structural features have significant implications for the properties of smectite, such as swelling and rheological behavior.

### 2.2. Rheology

Rheology is the science that studies the flow and deformation behavior of materials. It is commonly represented by the relationship between shear stress (σ) and shear rate ($\dot{\gamma }$). If shear stress is directly proportional to the shear rate, the material behaves as a Newtonian fluid, and the proportionality constant is the viscosity coefficient (η) [32,49]:

(1) $$\sigma \;=\;\eta \dot{\gamma }$$

If the relationship between shear stress and shear rate is nonlinear, the fluid is considered non-Newtonian, and the viscosity does not have a single value. In this case, the concept of apparent viscosity is used, which is defined as the slope of a line that passes through the origin and intersects the flow curve at a specific shear rate [50]:

(2) $$\eta_{a}\;=\;\frac{\sigma }{\dot{\gamma }}$$

where $\eta_{a}$ is the apparent viscosity at a given shear rate.

**Figure 3:**
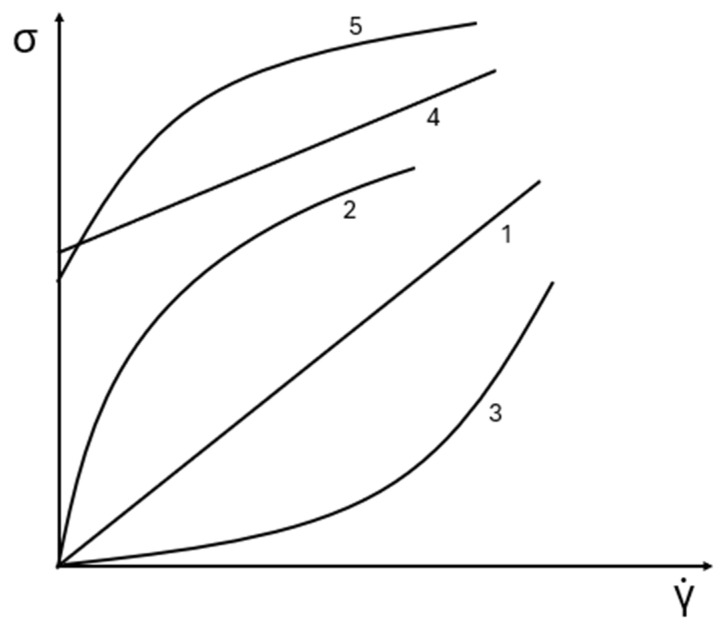
Typical curves relating shear stress to shear rate. (1) Newtonian behavior, (2) shear-thinning behavior, (3) shear-thickening behavior, (4) Bingham plastic behavior, and (5) non-Newtonian yield stress behavior. Adapted from [51].

Figure 3 illustrates typical flow curves that describe the relationship between shear stress and shear rate. Curve 1 represents Newtonian behavior. Curve 2 corresponds to a shear-thinning fluid, where shear stress increases less than proportionally with shear rate, leading to a decrease in apparent viscosity as shear rate increases.

Curve 3 shows the opposite behavior (shear-thickening), where viscosity increases with an increasing shear rate.

A power-law model can describe shear-thinning and shear-thickening behaviors [32,50]:

(3) $$\sigma \;=\;k{\dot{\gamma }}^{n}$$

where n is the flow behavior index. If n < 1, the fluid is pseudoplastic. The lower the n, the stronger the shear-thinning behavior [49].

As the shear rate approaches very low values, many materials exhibit a finite stress below which they do not flow. This stress is referred to as the yield stress. It represents the minimum shear stress required to initiate flow.

Curve 4 illustrates the behavior of a Bingham plastic, which behaves as a Newtonian fluid beyond a finite yield stress. The Bingham model is expressed as [50]:

(4) $$\sigma \;=\;\sigma_{y}^{B}\;+\;\eta_{\mathrm{pl}}\dot{\gamma }$$

where $\sigma_{y}^{B}$ is the Bingham yield stress and $\eta_{\mathrm{pl}}$ is the plastic viscosity.

Curve 5 represents a non-Newtonian yield stress fluid and is better described by the Herschel–Bulkley model [50]:

(5) $$\sigma \;={\;\sigma }_{y}^{H}\;+\;k{\dot{\gamma }}^{n}$$

where $\sigma_{y}^{H}$ is the Herschel–Bulkley yield stress.

Some suspensions exhibit time-dependent behavior in which viscosity is not solely a function of shear rate. In such systems, the stress response may not be instantaneous due to particle rearrangement or changes in internal structure. This leads to: (i) Thixotropy, where viscosity decreases over time at constant shear rate, and (ii) Rheopecty, where viscosity increases over time under constant shear. These behaviors are particularly important in complex fluids, such as clay-rich slurries, where microstructural evolution significantly impacts flow resistance.

### 2.3. Influence of Clays on the Rheology of Mineral Pulps

Suspensions containing clay minerals generally exhibit pseudoplastic behavior, in which the apparent viscosity decreases as the shear rate increases. The fine particle size and laminar morphology of clay minerals significantly alter interparticle interactions, leading to structural changes that directly impact the rheological behavior of the suspension.

**Figure 4:**
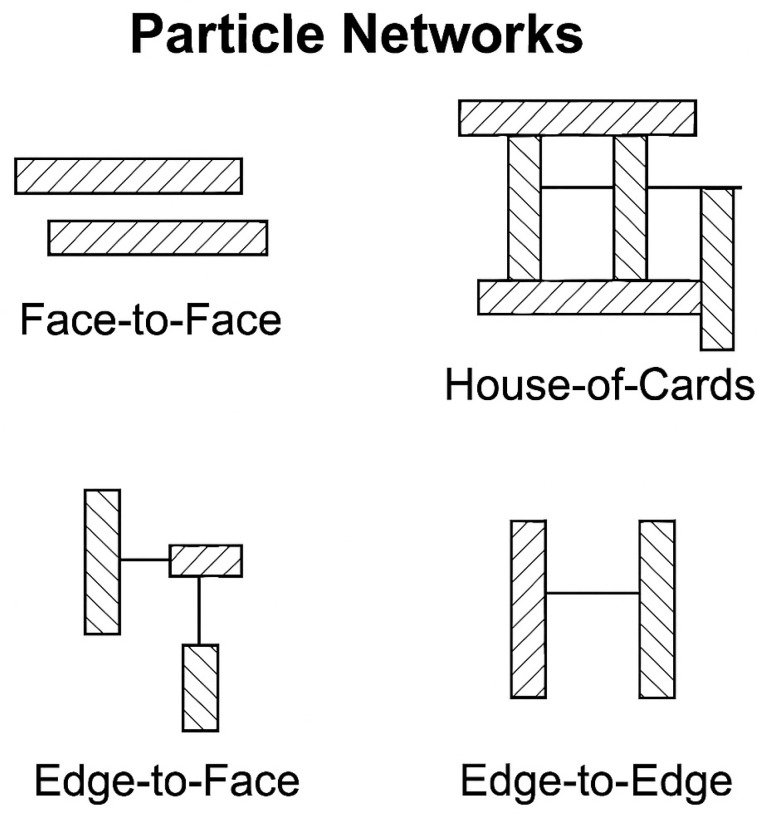
Representation of the aggregation modes between clay mineral particles.

The formation of particle networks is considered the main mechanism behind the increase in viscosity (Figure 4). These networks restrict the free movement of particles and induce gel-like structures that resist flow. Additionally, the swelling capacity of smectites in aqueous media further enhances viscosity due to the expansion of the mineral layers, which forms a three-dimensional structure within the fluid.

On the other hand, kaolinite-based suspensions lead to a less pronounced increase in viscosity compared to expansive clays. However, the viscosity of kaolinite suspensions tends to be more stable over time.

Hong et al. [52] investigated the rheological characteristics of kaolin–sand slurries as a function of pH, temperature, solid concentration, and mixing ratio. They found that a higher kaolin content increased viscosity, with erratic behavior observed when the solid concentration exceeded 50 wt%.

Nuntiya and Prasanphan [53] studied the rheological response of three types of kaolin (Narathiwat, Lampang, and Ranong) and reported that both viscosity and thixotropy increased with solid content. The Narathiwat kaolin showed the highest plastic viscosity, attributed to its well-defined hexagonal plates and smaller particle size.

**Figure 5:**
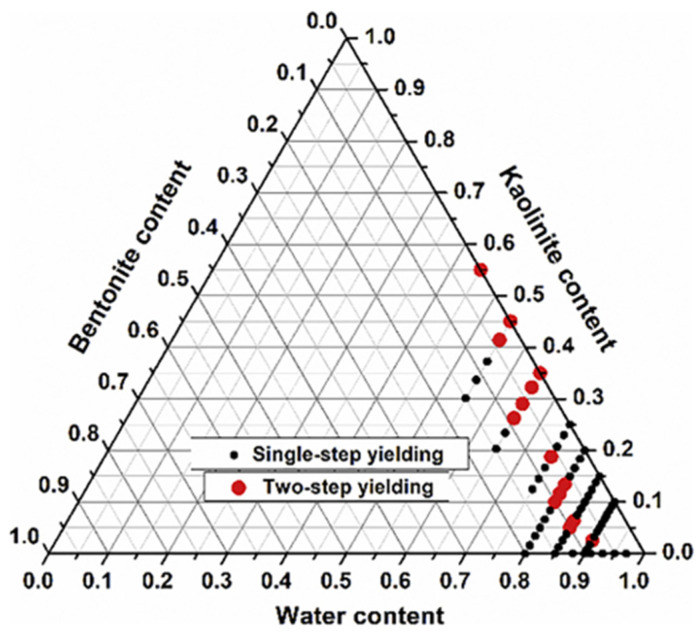
Ternary diagram for water/kaolinite/bentonite suspensions, black dots represent systems with single-step yielding and red dots represent systems having two-step yielding behavior. Adapted from [54].

Shakeel et al. [54] analyzed the rheology and yielding transitions in mixed kaolinite–bentonite suspensions using amplitude sweep, frequency sweep, stress ramp-up, and structural recovery tests. Kaolinite-rich suspensions exhibited two-step yielding behavior at high solid contents (35 wt%), while bentonite-rich suspensions showed single-step yielding even at high concentrations. A clear transition between one-step and two-step yielding was observed as a function of the kaolinite/bentonite ratio for a fixed total solid content (Figure 5). Single-step yielding refers to the transition of a material directly from an elastic state to a viscous flow regime upon surpassing a single stress threshold. In contrast, two-step yielding involves the presence of two distinct mechanical transitions: (i) the first associated with the breakdown of weaker structures (such as reversible or edge–face interactions), and (ii) the second corresponding to the disruption of a denser or more interconnected particle network, ultimately leading the material to flow.

Burdukova et al. [55] evaluated the relationship between rheology and mineralogical composition of three types of natural ores. Talc-rich samples showed the lowest impact on yield stress and Bingham viscosity. In contrast, serpentine-rich ores produced a dramatic increase in yield stress, even at low solids volume fractions. Smectite-rich ores had the greatest influence on Bingham viscosity.

Although all phyllosilicate groups significantly affected rheology, it remains unclear which type induces the most detrimental effect on mineral processing.

Crystallinity also plays a crucial role in the rheology of clay suspensions. Zhang and Peng [56] found that poorly crystalline kaolinite (Q38) induced greater viscosity than highly crystalline kaolinite (Snobrite). Q38 exhibits more disordered edges and a complex basal surface, whereas Snobrite displays thicker edge dimensions and a more ordered structure [57].

**Figure 6:**
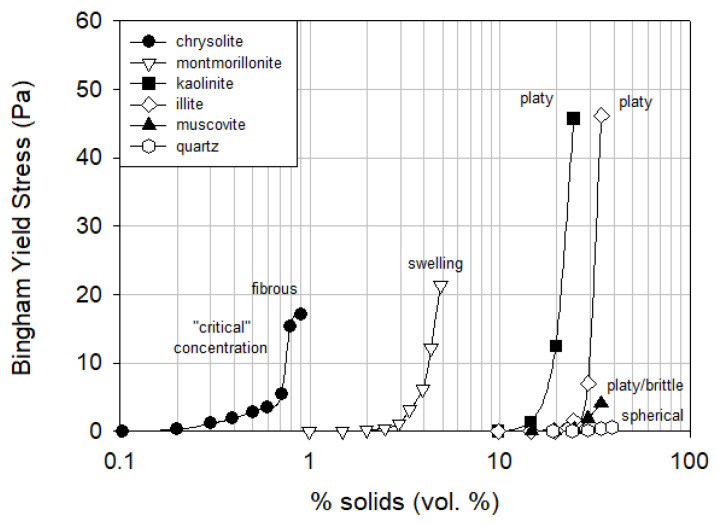
A comparison of the Bingham yield stresses of phyllosilicate group minerals. Redrawn from [58].

Ndlovu et al. [58] conducted a comparative study of the rheological behavior of different phyllosilicate groups. Figure 6 shows the Bingham yield stress of each mineral as a function of solids volume fraction. A critical concentration was identified, beyond which yield stress increased exponentially, indicating a threshold at which rheology may become unmanageable in processing operations. Chrysotile (serpentine group) showed the most severe behavior with a critical concentration as low as 0.7 vol%. Montmorillonite (a member of the smectite group) exhibited a critical point near 4 vol%, attributed to its high swelling capacity. Other minerals exhibited higher critical concentrations, with differences linked to charge anisotropy, swelling behavior, crystallinity, and surface morphology.

## 3. Rheology-Modifying Reagents

The rheological behavior of mineral suspensions containing clays is strongly influenced by the addition of chemical reagents that induce particle dispersion or aggregation phenomena. This section focuses exclusively on reagents that have been studied in clay-rich systems, particularly those containing kaolinite, montmorillonite, bentonite, and illite. The physicochemical characteristics of these minerals—such as surface charge heterogeneity, swelling capacity, and colloidal size—determine their interaction mechanisms with salts, pH modifiers, polymers, surfactants, and nanoparticles.

Subsequently, each subsection examines how these reagents alter the rheological properties of clay suspensions, emphasizing their interaction mechanisms with phyllosilicates and the effects on viscosity, yield stress, and structural behavior. Where appropriate, examples from the literature have been selected or reinterpreted to highlight their relevance in mineral systems containing clays.

### 3.1. Salinity and pH

The adsorption of ionic species onto the surface of dispersed particles in aqueous media can alter their degree of dispersion or coagulation. These ions modify the net surface charge of the particles, thereby influencing interparticle interactions. In aqueous suspensions, these ions are commonly introduced through inorganic additives, such as salts or pH modifiers, to adjust the rheological behavior.

Tombácz and Szekeres [59] found that the rheology of a 4 wt% montmorillonite suspension changed from Newtonian to pseudoplastic or mildly thixotropic as pH decreased in the presence of 0.01 M NaCl. A sharp increase in Bingham yield stress was observed below pH 7, indicating the formation of a stronger gel structure. This finding is consistent with earlier results by Durán et al. [60]. The decrease in pH in the presence of NaCl induces a progressive gelation of montmorillonite suspensions, attributed to the enhanced electrostatic attractions between oppositely charged faces and edges, which increases the yield stress.

**Figure 7:**
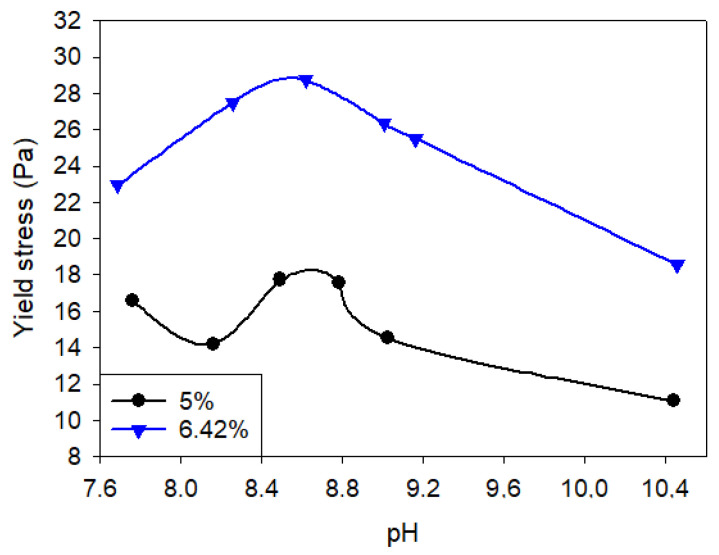
Variation of yield stress ($\sigma_{y}$) with pH at the two bentonite concentrations. Natural pH of 5% was 8.9, and of 6.42% was 9.1. Redrawn from [39].

Kelessidis et al. [39] reported an optimal pH of 8.7 for a 6.42 wt% bentonite suspension, slightly lower than its natural pH of 9.1, where the highest Herschel–Bulkley yield stress was achieved. A similar pH optimum (8.6–8.9) was observed for a 5 wt% bentonite suspension with a natural pH of 8.9. In both cases, yield stress decreased monotonically at pH values outside this optimum (Figure 7). Notably, the type of pH modifier used can result in different viscosity values, even at the same target pH. The viscosities of a bentonite suspension adjusted to pH 10.5 at a shear rate of 160 s^−1^ followed the following order: η_NaOH_ > η_Ca(OH)_2__ > η_Na_2_CO_3__ [61].

**Figure 8:**
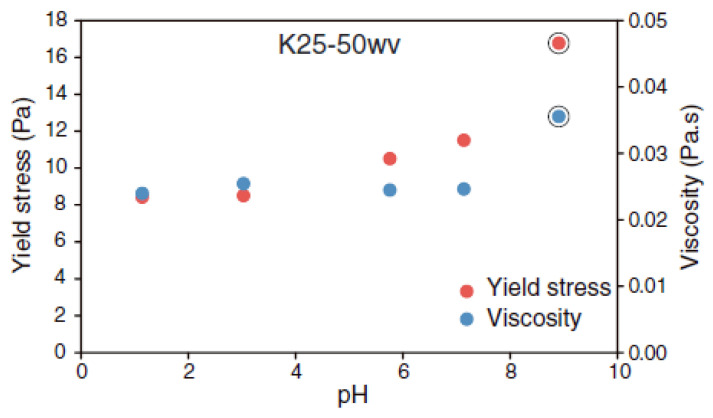
Variations in yield stress and viscosity of smectite-quartz suspensions with pH. Adapted from [62].

In contrast, Kameda and Morisaki [62] observed that both yield stress and dynamic viscosity of a smectite–quartz suspension (25 wt% smectite, 50% *w*/*v*) gradually decreased with decreasing pH (Figure 8). This behavior can be attributed to the presence of quartz, whose surface charge shifts from positive to negative near a pH of 2–3. Under acidic conditions, the electrostatic attraction between nearly neutral or positively charged quartz and negatively charged smectite could hinder edge–face interactions among smectite particles, weakening the resulting structure and reducing yield stress.

**Figure 9:**
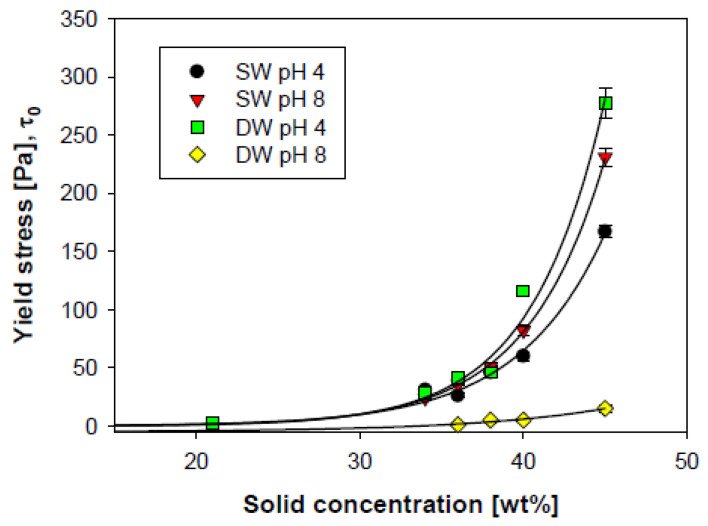
Yield stress of kaolin slurries prepared in seawater (SW) and distilled water (DW). Adapted from [64].

Other studies have shown that the yield stress of kaolinite suspensions varies with both pH and salinity. Nasser and James [63] reported yield stress values for 10 vol% kaolinite suspensions across different ionic strengths and pH levels. Yield stress decreased with increasing ionic strength at low pH, but increased with ionic strength at high pH. Leiva et al. [64] observed similar trends using kaolin suspensions at varying concentrations in both distilled water and seawater, at pH levels of 4 and 8. In seawater, increasing the pH from 4 to 8 resulted in a slight increase in yield stress, whereas in distilled water, the same pH shift led to a substantial increase (Figure 9). In low-pH, electrolyte-free conditions, kaolin particle edges are highly positively charged, while the faces retain a constant negative charge. This promotes strong edge–face interactions and aggregation. Increasing electrolyte concentration compresses the electrical double layer and reduces electrostatic repulsion, thereby weakening these interactions. At high pH, face–to–face interactions dominate, which are weaker than edge–to–face associations in deionized systems, but become stronger in high ionic strength environments.

The effect of electrolytes on rheology depends on the type, concentration, charge, and valence of the ions. Ions alter the surface charge of particles, which governs their tendency to agglomerate or disperse. Surface charge can be quantified through zeta potential measurements. A high absolute value of zeta potential favors dispersion, while values closer to zero promote aggregation. The former reduces rheological resistance, whereas the latter increases it [65,66,67].

The literature indicates that the effects of salts and pH modifiers on the rheological properties of clay mineral suspensions yield inconsistent results. On the one hand, several studies report the effective compression of the electrical double layer and alteration of the particle surface charge. On the other hand, some studies show unpredictable rheological behavior under certain saline concentration or pH conditions. Moreover, most available studies are conducted under controlled conditions (e.g., pure clay minerals in deionized water or predefined salinity), which may differ from the responses observed in real multicomponent suspensions that exhibit mineralogical and saline heterogeneity. As such, these variations may alter the effectiveness of these reagents. Therefore, detailed geochemical characterizations of real suspensions, along with systematic studies, are necessary to establish the optimal operational efficiency of each reagent. Furthermore, potential synergistic or antagonistic effects between salts and other rheological modifiers, such as polymers or nanoparticles, warrant in-depth investigation.

### 3.2. Polymers

Polymers can interact with clay particles through various mechanisms: (i) Ionic polymers are adsorbed onto the particle surface via electrostatic interactions, while (ii) Nonionic polymers act primarily through steric interactions. The effectiveness of polymer–particle interactions depends on the physicochemical properties of both components, including the molecular weight, functional groups, and concentration of the polymer, as well as the size, morphology, and surface charge of the particles.

Among the most widely studied reagents is sodium polyacrylate (NaPA), which has demonstrated effectiveness as a rheology modifier for clay-rich suspensions. Low molecular weight NaPA is a successful dispersant for laponite suspensions in low ionic strength media [68], and for kaolinite or quartz–clay mixtures in seawater (high ionic strength) [69,70]. NaPA absorbs clay particles, forming a steric barrier that prevents agglomeration.

**Figure 10:**
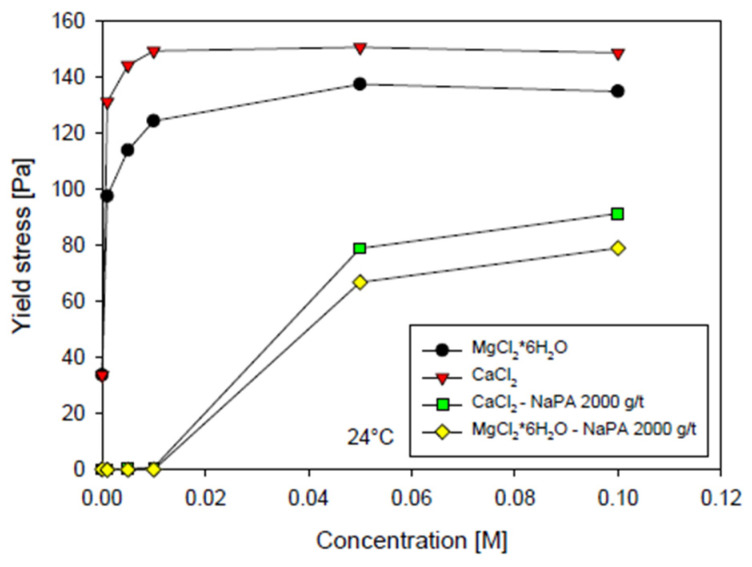
Yield stress as a function of the molar concentration of Ca^2+^ and Mg^2+^ in the absence and presence of NaPA. Adapted from [71].

Ramos et al. [71] observed that the dispersing efficiency of NaPA in kaolin–quartz suspensions decreased in the presence of divalent cations (Ca^2+^ and Mg^2+^), which interfere with polymer adsorption. Molecular dynamics simulations suggest that polymer adsorption is influenced by both the type of mineral and the nature of the ions. On quartz surfaces, calcium promotes stronger adsorption, while magnesium has a greater affinity for kaolinite (Figure 10). NaPA is a linear anionic polymer with carboxylate groups (–COO^−^) distributed along its backbone, which, through the repulsion between polymer layers adsorbed onto adjacent particles, prevents their approach to distances at which attractive forces become significant [70,72].

Rossi et al. [73] and Gareche et al. [74] investigated the effects of low molecular weight polymers on the rheology of bentonite suspensions, with divergent results. Rossi et al. found that increasing concentrations of nonylphenol–polypropylene oxide–polyethylene oxide block copolymer (NPEB) increased yield stress, reaching a maximum at ~50% bentonite surface coverage. Further polymer addition reduced yield stress, but values remained higher than those of polymer-free suspensions. This increase is explained by physical interactions of the bridging flocculation type between bentonite particles, induced by the copolymeric structure of NPEB. Conversely, Gareche et al. observed that polyethylene oxide (PEO) improved suspension stability by reducing yield stress, attributed to favorable interactions with bentonite particles. PEO acts as a non-ionic dispersant, stabilizing the suspension through a steric repulsion layer that reduces attractive interactions between particles.

Several studies have also investigated the impact of polymer concentration and molecular weight on the rheology of clay suspensions.

**Figure 11:**
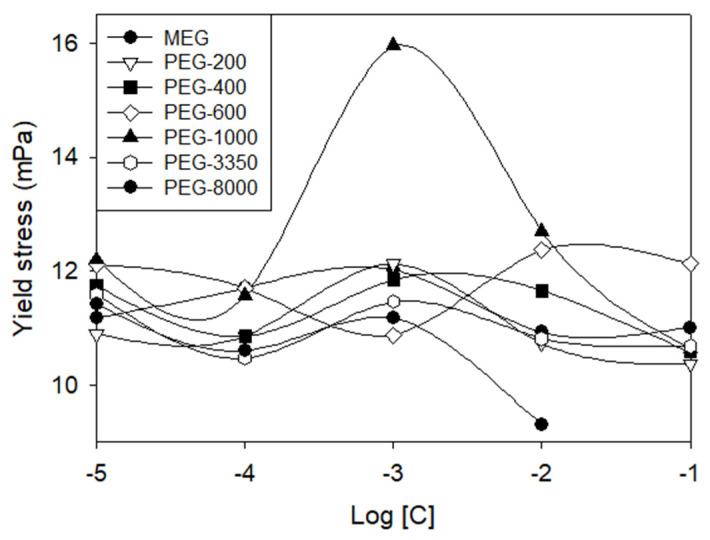
Yield stress values as a function of PEG concentration. Redrawn from [75].

Tunc and Duman [75] evaluated monoethylene glycol (MEG) and polyethylene glycol (PEG) at various molecular weights and concentrations in Na-bentonite suspensions. Their results showed no consistent trend: yield stress either increased or decreased depending on specific combinations of polymer properties (Figure 11). The rheological effects of MEG and PEG on bentonite result from a complex balance between surface adsorption, molecular weight, and the conformational structure of the polymer in solution.

Kim and Carty [76] identified an optimal molecular weight for polyacrylic acid (PAA) that minimized the apparent viscosity of Huntingdon clay suspensions. At higher concentrations, viscosity increased, with the extent of the increase depending on the molecular weight. The behavior of PAA reflects a transition between electrostatic dispersion (at low molecular weight/concentration) and bridging flocculation (at high molecular weight/concentration).

Conceição et al. [77] reported that both yield stress and viscosity of kaolin suspensions increased with increasing concentration and molecular weight of carboxymethyl cellulose (CMC). These results suggest that CMC molecules may remain in solution rather than adsorbing onto kaolinite surfaces. CMC increases the viscosity of the system primarily by modifying the continuous phase, rather than through direct interaction with kaolinite particles.

The functional groups of polymers determine their ionic character and influence their rheological performance. For instance, nonionic polyvinyl alcohol (PVA) modified the plastic viscosity of clay suspensions differently depending on the clay type [78]. The influence of polycarboxylate ether (PCE) on 35 wt% kaolinite suspensions was studied under acidic and alkaline conditions [79]. In acidic media, both yield stress and apparent viscosity (at 10 s^−1^) showed a moderate peak; under alkaline conditions, a much sharper peak was observed. PCE chains remain neutral in acid but become negatively charged and extended in alkaline solutions. Thus, interactions with kaolinite can occur via hydrogen bonding (side chains) or electrostatic interactions (main chains), depending on pH.

**Figure 12:**
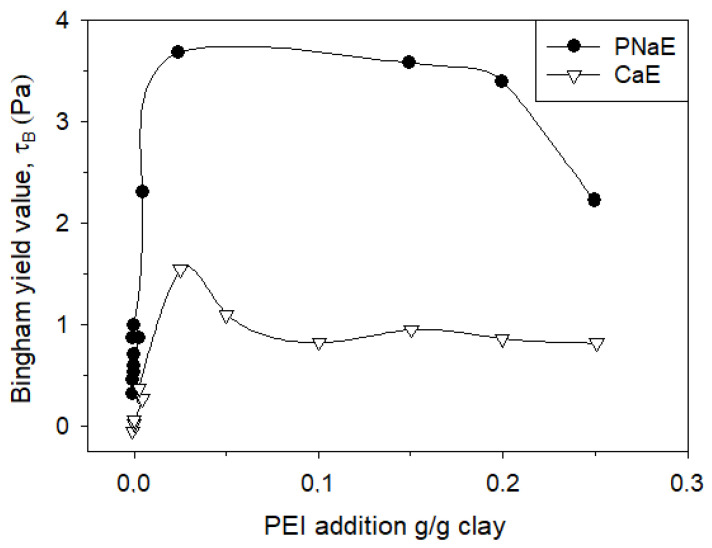
Bingham yield value of the CaE and PNaE dispersions. Redrawn from [79].

Alemdar et al. [79] reported the effects of polyethyleneimine (PEI), a cationic polyelectrolyte, on purified bentonite suspensions. Yield stress varied with PEI concentration (g PEI/g clay), reflecting the influence of bentonite mineral structure on rheological response (Figure 12). PEI interacts primarily through electrostatic adsorption and interparticle bridging, modulating the suspension’s rheology in response to changes in polymer concentration and mineral characteristics. In another study, polyanionic cellulose (PAC) was shown to mitigate the adverse effects of ionic strength on bentonite suspension viscosity [80]. PAC enhances rheological stability under high salinity conditions through a combination of partial adsorption, steric repulsion, and increased viscosity of the dispersing medium.

Biopolymers have also been explored as eco-friendly alternatives for modifying the rheology of bentonite-based drilling fluids, due to their biodegradability, non-toxicity, and renewable origin. Some biopolymers are used in their natural form [81,82], while others require chemical modification to meet the demanding conditions of water-based drilling fluids [83,84]. For example, sugarcane bagasse may improve rheological properties due to its long fibers, while raw sugarcane fibers have been shown to reduce viscosity at high shear rates significantly [85].

Chitin is one of the most abundant natural polysaccharides, and chitosan, its deacetylated derivative, is a biodegradable, biofunctional, and renewable polymer. However, the poor solubility of chitin limits its application [86,87].

**Figure 13:**
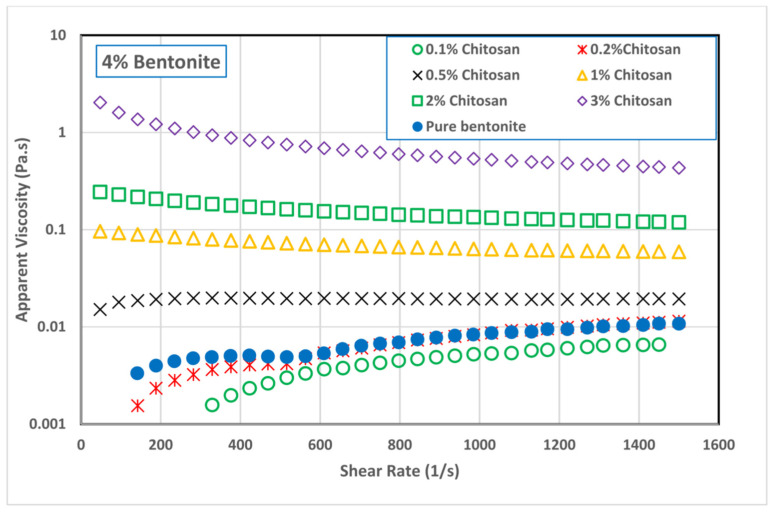
Effect of chitosan concentration on the viscosity of 4.0 wt% bentonite dispersion. Adapted from [88].

Abu-Jdayil et al. [88] evaluated natural chitosan as a rheological modifier in Na-bentonite-based drilling fluids. At certain chitosan concentrations, the viscosity increased sharply due to the formation of a network. At lower concentrations, the suspension exhibited a more dispersed character (Figure 13). Simultaneous increases in both chitosan and bentonite content promoted gel formation and significantly elevated yield stress.

Numerous natural additives and their derivatives have been investigated as rheology modifiers in bentonite-based fluids. Examples include grass fibers [89], nanocellulose [90], carrageenans [91], rice husk [92], protein isolates [93], and henna extracts [94].

Gao et al. [95] studied basil seed powder (BSP) as an additive in sodium bentonite-based fluids. Viscosity increased with BSP concentration, especially above 1 wt%. The apparent viscosity and yield stress increased almost linearly below 1 wt%, but rose sharply beyond that threshold. At 3 wt%, rheological behavior was deemed excessively high.

Nwosu and Ewulonu [96] evaluated eco-friendly drilling fluids based on deionized water–bentonite suspensions modified with three biopolymers: carboxymethyl cellulose (CMC), xanthan gum (Xanplex D), and polyanionic cellulose (PAC-R). PAC-R displayed the highest shear rate and shear stress values, attributed to its extended linear chain structure and enhanced interaction with water and solids. It also served as the most effective viscosifier due to its strong negative charge.

The rheological response following polymer addition strongly depends on molecular weight and architecture, ionic character, dosage, and mineral type, offering considerable versatility. For instance, high-molecular-weight polymers tend to promote flocculation via bridging mechanisms, thereby enhancing rheological properties or facilitating sedimentation. In contrast, low-molecular-weight polymers typically act as dispersants, reducing rheological properties through steric repulsion. Regarding architecture, linear polymers facilitate extensive adsorption onto particle surfaces, whereas branched or comb-like polymers may form more compact or gel-like structures. Additionally, low dosages may be insufficient to induce significant effects, while excessive dosages may lead to overstabilization or even re-aggregation due to the saturation of active sites. Finally, the polymer’s ionic character determines its interaction with clays: anionic polymers, such as sulfonated polyacrylamide, exhibit a strong affinity for positively charged particles (e.g., certain metallic impurities), while cationic polymers tend to adsorb strongly onto negatively charged surfaces, such as montmorillonite, thereby promoting flocculation.

### 3.3. Surfactants

In industrial processes such as wastewater treatment, soil remediation, enhanced oil recovery, mineral flotation, and ceramic manufacturing, the adsorption of soluble surfactants onto the surfaces of solid particles in concentrated colloidal dispersions is essential. For instance, in the ceramic industry, controlling the colloidal stability and properties of particle suspensions is crucial to achieving high-quality products. Therefore, the surfactant adsorption process must be carefully controlled to ensure satisfactory performance [97,98,99].

Surfactants are characterized by their amphiphilic molecular structure, comprising a hydrophilic (water-attracting) head and a hydrophobic (water-repelling) tail. They are further classified by their ionic nature—anionic, cationic, or nonionic—and molecular weight. Surfactants can reduce surface tension, enhance particle dispersion, and modify suspension structure, all of which influence rheological behavior.

Numerous studies have investigated the role of surfactants as rheology modifiers in clay suspensions. For example, the addition of various surfactants has led to a wide range of effects in bentonite-based systems [40,100,101,102]. Among the most studied factors is the ionic character of surfactants.

Günister et al. [103] examined the adsorption, bridging, and intercalation effects of a cationic surfactant, benzyldimethyltetradecyl ammonium chloride (BDTDACl), on bentonite suspensions. Increasing surfactant concentration changed the fluid behavior from Newtonian to pseudoplastic and introduced a yield stress. An optimal surfactant concentration was identified, at which yield stress was maximized. Cationic surfactants, such as BDTDACl, can adsorb onto the negatively charged surfaces of minerals such as montmorillonite or kaolinite, thereby reducing electrostatic repulsion and promoting flocculation through charge neutralization and hydrophobic bridging.

**Figure 14:**
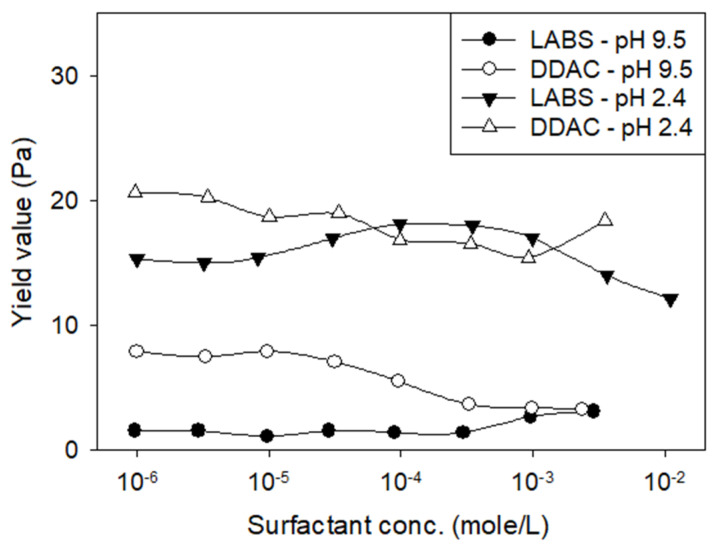
Yield value of the sodium bentonite slurries (6% *w*/*w*) versus the amount of DDAC and LABS. Redrawn from [44].

Güngör [44] studied the rheological behavior of sodium-activated bentonite after adding an anionic surfactant (linear alkyl benzene sulfonate, LABS) and a cationic surfactant (distearyl dimethyl ammonium chloride, DDAC) at various concentrations (Figure 14): (i) at pH 9.5, LABS increased yield stress with increasing concentration, while DDAC reduced it, (ii) at pH 2.4, LABS exhibited a maximum yield stress at an optimal concentration, whereas DDAC showed a minimum yield stress at a different concentration.

**Figure 15:**
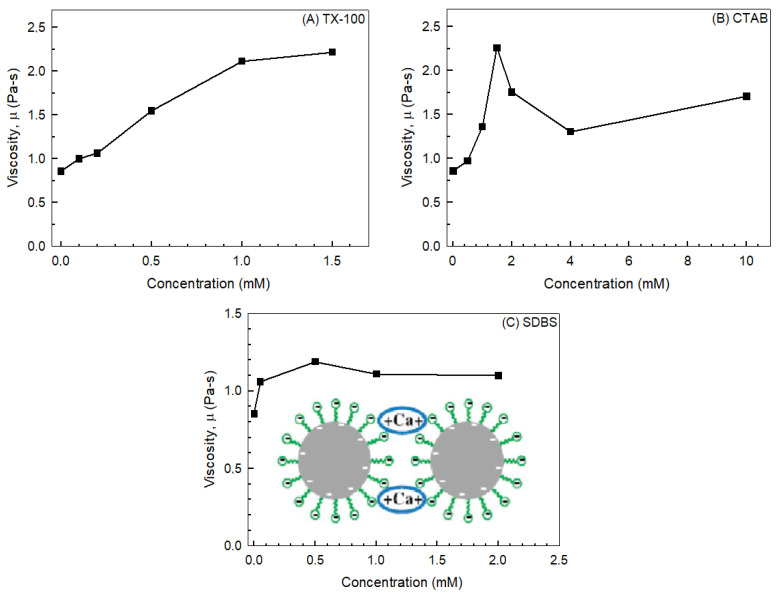
Viscosity of 55% pyrophyllite-water slurry at a 60 s^−1^ constant shear rate in the presence of (**A**) TX-100, (**B**) CPB, and (**C**) SDBS. Inset of c shows the schematic presentation of bridging of two clay particles in the presence of SDBS and a Ca^2+^ ion. Redrawn from [104].

In contrast, anionic surfactants such as sodium dodecylbenzene sulfonate (SDBS) can increase electrostatic repulsion between particles when they carry negative charges, leading to enhanced dispersion and reduced viscosity. However, the presence of divalent cations (Ca^2+^, Mg^2+^ can mediate bridged interactions between the sulfonate group and mineral surfaces, thereby promoting aggregate formation, as demonstrated by Desai et al. [104]. These authors investigated the rheology of pyrophyllite–water slurries in the presence of anionic, cationic, and nonionic surfactants (Figure 15). At 55 wt% solids and a shear rate of 60 s^−1^, viscosity was measured for: (i) Triton X-100 (TX-100), a nonionic surfactant, which increased viscosity with concentration; (ii) Cetylpyridinium bromide (CPB), a cationic surfactant, which increased viscosity up to a certain concentration, then decreased, and later increased again; (iii) Sodium dodecylbenzene sulfonate, an anionic surfactant, which increased viscosity at low concentrations but produced negligible changes at higher levels. The presence of divalent ions such as Ca^2+^ facilitated particle bridging, enhancing the rheological effects of SDBS.

Other studies have examined the influence of cationic and anionic surfactants [105,106,107,108], cationic surfactants alone [109,110,111], and nonionic surfactants [112]. Non-ionic surfactants, in turn, act primarily through steric interactions. These surfactants form adsorbed layers that induce volume exclusion, thereby affecting the viscosity and structure of the suspension, as evidenced in studies involving Triton X-100 and mixtures with sodium polyacrylate (NaPA), a negatively charged polymer.

**Figure 16:**
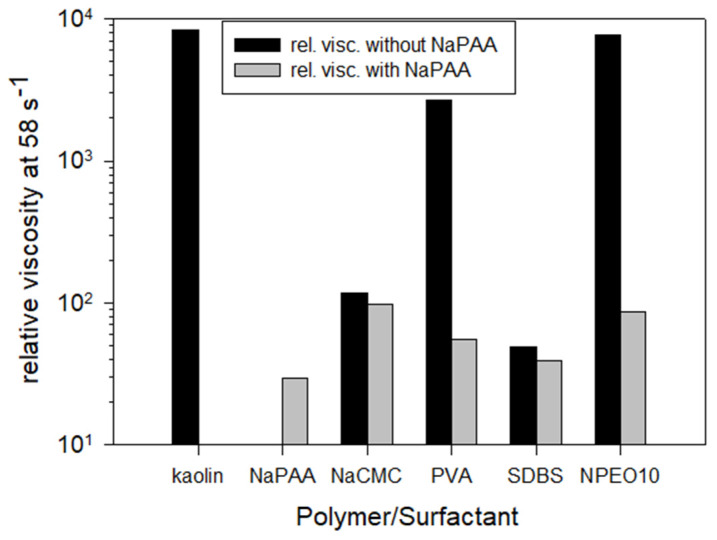
Relative viscosity for 60 wt% kaolin dispersions at 58 s^−1^, with addition of different polymers/surfactants, in presence and absence of NaPAA. Redrawn from [113].

An interesting study by Sjöberg et al. [113] investigated the effect of adding surfactants and polymers to kaolin suspensions, both with and without sodium polyacrylate (NaPAA) pretreatment. In the absence of NaPAA, all other additives reduced the relative viscosity of the kaolin suspension to varying degrees. When NaPAA was used as a dispersant before adding other additives, viscosity decreased even further (Figure 16). This suggests that the combined action of multiple reagents can produce a more pronounced rheological modification than the action of individual components.

The behavior of surfactants in the rheological properties of clay mineral suspensions is highly sensitive to their ionic character, dosage relative to the critical micelle concentration (CMC*), and the mineralogical composition of the suspension. For example, cationic surfactants such as CTAB tend to adsorb strongly onto negatively charged clay surfaces (e.g., montmorillonite), promoting flocculation via charge neutralization or electrostatic bridging. In contrast, anionic surfactants (e.g., SDS) can enhance electrostatic repulsion between similarly charged clay particles, leading to increased dispersion and reduced rheological strength. Non-ionic surfactants primarily act through steric and hydration effects, stabilizing the suspension without significantly altering the surface charge, although their behavior may vary with temperature and the ionic strength of the medium.

Regarding the concentration relative to the CMC*, this has not been explicitly addressed in the reviewed studies on clay suspensions, despite being a well-recognized phenomenon in colloidal systems [114,115,116,117,118]. Below the CMC*, surfactants adsorb as monomers onto mineral surfaces, modifying particle–particle interactions; above the CMC*, micelles begin to form in solution, which may reduce the availability of free surfactant for adsorption or even induce aggregation through alternative mechanisms, such as dehydration of adsorbed layers.

Finally, minerals with high specific surface charge or cation exchange capacity (e.g., smectites or illites) exhibit greater sensitivity to surfactants than less reactive minerals such as kaolinite or pyrophyllite. This variability determines whether the net effect of the surfactant will be flocculation, stabilization, or even the formation of a gel structure. While this phenomenon has not been addressed in the reviewed studies, the colloidal science literature suggests that the sensitivity of clays to surfactants depends on their surface charge, and cation exchange capacity [119,120,121].

### 3.4. Nanoparticles

Until recently, synthetic and natural polymers, as well as surfactants, have been widely used as rheology modifiers. However, the rise in nanotechnology has expanded the range of available additives in fields such as engineering, medicine, energy, and environmental science. The exceptionally high surface-to-volume ratio of nanomaterials enables superior or even unexpected performance in systems dominated by surface effects.

Nanoparticles (NPs), typically ranging from 1 to 100 nanometers in size, possess significantly higher specific surface area than their micro-sized counterparts. They can be characterized by size, surface charge, and morphology [122,123,124,125,126,127]. The addition of nanoparticles such as graphene [128], carbon nanotubes [129], silica [130], and metal oxides [131,132] has been extensively studied in bentonite-based water drilling fluids. Even low NP concentrations (typically <1.0 wt%) have shown remarkable improvements in rheological properties. The use of nanoparticles as rheological modifiers involves mechanisms such as heteroflocculation, electrostatic interactions, colloidal bridging, surface adsorption, and structural modification of mineral networks.

**Figure 17:**
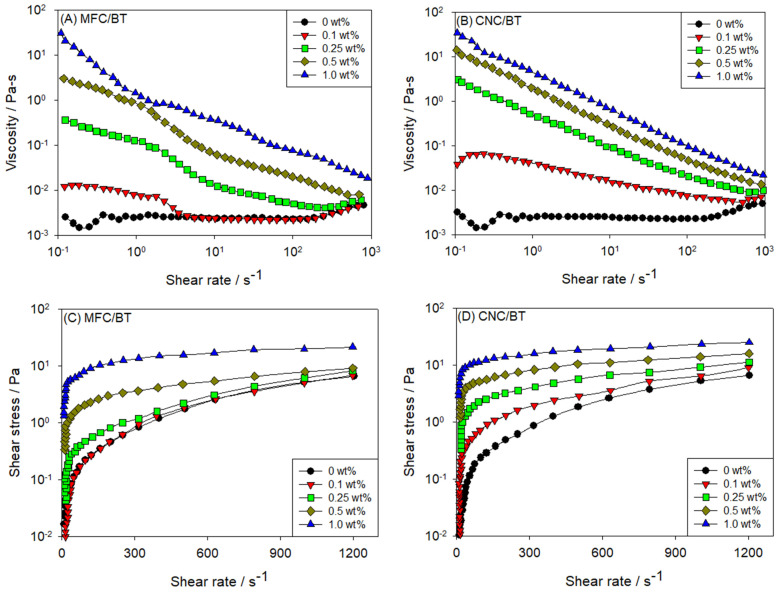
Plots of viscosity versus shear rate for (**A**) MFC/BT-, and (**B**) CNC/BT-WDFs. Shear stress versus shear rate for (**C**) MFC/BT-, and (**D**) CNC/BT-WDFs at different CNP concentrations: 0 wt% (black circles), 0.1 wt% (red inverted triangles), 0.25 wt% (green squares), 0.5 wt% (dark yellow diamonds), and 1.0 wt% (blue triangles). (Dash lines in panels c and d represent the fitted lines using the Herschel−Bulkley model). Redrawn from [133].

Cellulose-based nanoparticles have received growing interest due to their abundance and renewable nature. Mei-Chun Li et al. [133] demonstrated the effectiveness of cellulose nanoparticles (CNPs)—including microfibrillated cellulose (MFC) and cellulose nanocrystals (CNCs)—in enhancing the rheological and filtration performance of bentonite-based drilling fluids. CNCs outperformed both MFCs and NP-free systems by providing superior rheology, higher temperature stability, reduced fluid loss, and thinner filter cakes (Figure 17).

**Figure 18:**
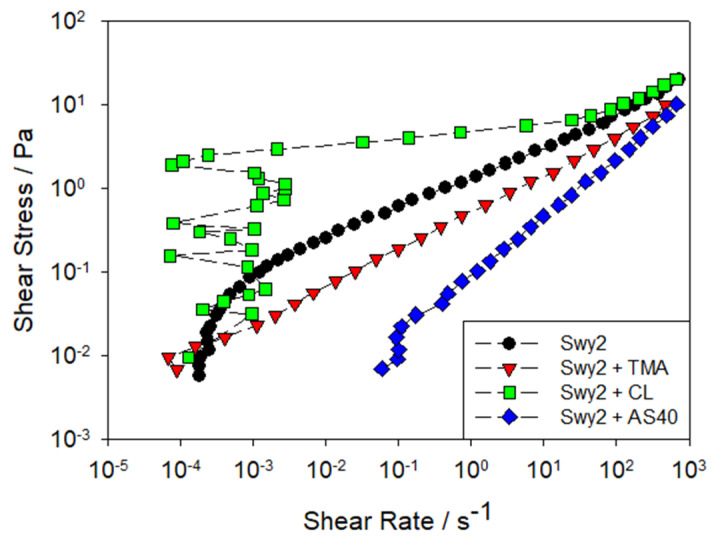
Controlled stress steady shear rheology of 2.4 wt% montmorillonite-silica suspensions. For each sample, the open symbols represent the ramp-down data. Redrawn from [134].

Improved rheological properties in clay-based suspensions can also be achieved using cost-effective and readily available materials. For this reason, numerous studies have focused on silica-based nanoparticles, including: (i) cationic and anionic silica added to montmorillonite suspensions [134], (ii) silica nanospheres of various sizes in kaolin suspensions [135], (iii) PEG-coated silica nanoparticles in montmorillonite suspensions [136], (iv) anionic silica nanospheres in hectorite gels [137]. Bailey et al. [134] investigated the impact of spherical silica nanoparticles on the rheology of montmorillonite dispersions. Anionic silica types (Ludox AS40 and TMA) significantly reduced effective viscosity, with smaller AS40 particles having the strongest effect. In contrast, the cationic silica (Ludox CL) increased viscosity (Figure 18). Anionic silica nanoparticles induce colloidal stabilization through electrostatic repulsion when added to systems containing negatively charged montmorillonite, thereby reducing the effective viscosity of the system. This dispersion is also influenced by particle size, as smaller nanoparticles promote greater surface coverage, thereby intensifying the rheological effect.

Recent research also highlights the impact of different NPs on bentonite suspensions.

Vryzas et al. [138] examined the effects of commercial ${\mathrm{Fe}}_{3}O_{4}\;and\;\mathrm{Si}O_{2}$ nanoparticles, as well as custom-made bare and citric acid-coated ${\mathrm{Fe}}_{3}O_{4}$, on the rheology of bentonite slurries under alkaline conditions. All ${\mathrm{Fe}}_{3}O_{4}$ NPs increased yield stress, whereas $\mathrm{Si}O_{2}$ NPs decreased it. The authors suggested that attractive magnetic forces between ${\mathrm{Fe}}_{3}O_{4}$ particles may suppress electrostatic repulsion and promote aggregation, thus modifying rheological behavior.

Salih and Bilgesu [139] studied the rheology and filtration behavior of water-based drilling fluids containing anionic nanoparticles of silica, titanium, and aluminum at high pH (11.5–12). A 0.1 wt% dose of nanosilica significantly reduced yield stress, with diminishing returns at higher concentrations. A similar trend was observed for nanoaluminium and nanotitanium, although 0.3 wt% nanoaluminium proved more effective than nanotitanium.

**Figure 19:**
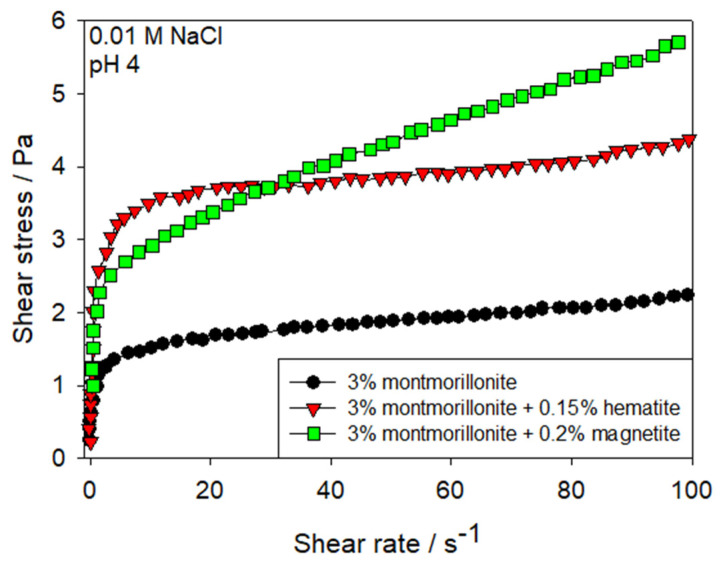
Flow curves for montmorillonite suspensions at pH 4 and low salt concentration in the presence of iron oxide nanoparticles. Redrawn from [141].

Nanoparticle morphology also plays a crucial role in determining the rheological behavior. Studies have examined spherical, cylindrical, and plate-like NPs. Spherical morphologies include: (i) iron oxide nanoparticles (magnetite and hematite) in montmorillonite suspensions (Figure 19), which enhance rheology by forming heteroaggregated networks with oppositely charged clay particles [140,141]; (ii) positively charged alumina-coated silica in ribbon-like hectorite suspensions [142]; (iii) anionic silica NPs in hectorite, montmorillonite, and beidellite suspensions [134,137,142,143]; (iv) silica-coated magnetic maghemite and cobalt ferrite NPs in Laponite suspensions, which shift the gelation threshold to lower Laponite concentrations [144,145,146]. Cylindrical morphologies have been explored using positively charged boehmite nanoparticles in hectorite suspensions [142], and in montmorillonite suspensions under monovalent salt and high-pH conditions [147]. Plate-like morphologies have been investigated through the synthesis of hydrotalcite nanoparticles added to montmorillonite suspensions [148,149,150]. These studies show that mixing positively charged hydrotalcite with negatively charged montmorillonite induces heteroflocculation, which increases viscosity at low shear rates and promotes the formation of gel-like networks resulting from the intercalation between layers of expansive minerals, thereby affecting the orientation and three-dimensional structure of the suspension. Time-dependent viscosity changes under high shear were also observed, indicating thixotropic behavior associated with the small size and structure of hydrotalcite particles.

Nanoparticles exhibit high potential as rheological modifiers due to their ability to interact with mineral surfaces, particularly through their large surface area. However, their effectiveness varies depending on nanoparticle type, surface charge, size, morphology, and chemical functionalization. For example, iron oxide nanoparticles may increase yield stress by suppressing electrostatic repulsion via attractive magnetic forces. In contrast, silica nanoparticles tend to form weaker structures, resulting in lower shear stress values. Positively charged nanoparticles can adsorb strongly onto negatively charged clay particles (such as montmorillonite), promoting flocculation via charge neutralization. Conversely, anionic nanoparticles may induce electrostatic repulsion, contributing to dispersion.

Lastly, the literature reveals numerous studies investigating the effect of chemical additives on the rheological properties of solid–liquid suspensions. However, most of these do not reflect the current physicochemical conditions of real industrial suspensions. According to Assefa and Kaushal [151], the function of chemical additives is often highly specific, with some additives being patented products designed for narrow applications that perform well in certain systems but are unsuitable for others. Therefore, the development of reagents with physicochemical properties tailored to specific application systems will be essential for achieving the desired or optimal rheological performance.

To summarize, Table 1 details the main types of reagents (salts, polymers, surfactants, nanoparticles), their mode of action, observed rheological effects (dispersion, flocculation, viscosity change, etc.), and relevant conditions (pH, salinity).

Table 2 compares the main advantages and limitations of the reviewed reagents used as rheological modifiers of clay mineral suspensions, highlighting their mechanisms of action, reported performance, and practical considerations for industrial implementation.

Unlike simple inorganic reagents (salts and pH modifiers), which primarily act by reducing electrostatic repulsion between particles, polymers offer greater functional flexibility, as they can be formulated as either dispersants or flocculants depending on their structure. Their effect can be tuned through parameters such as molecular weight, ionic charge, or branched architecture.

Surfactants, on the other hand, exhibit versatile effects depending on their type (anionic, cationic, or non-ionic), enabling aggregation or dispersion via surface adsorption or micelle formation mechanisms. However, their behavior can be highly nonlinear in relation to the concentration relative to the critical micelle concentration, and they may pose collateral issues, such as foam formation or environmental toxicity.

Nanoparticles represent an emerging class of high-performance additives due to their large surface-to-volume ratio, which allows for the adjustment of rheological microstructure through specific adsorption, network formation, or modification of clay packing arrangements. Nevertheless, their industrial application faces challenges related to cost, the need for colloidal stabilization, and environmental assessment.

### 3.5. Comparison of Rheological Behavior According to Clay Type

The rheological behavior of mineral suspensions modified by chemical additives varies significantly depending on the type of clay present, due to structural, morphological, and surface charge differences among kaolinite, montmorillonite, and illite:

(i) Montmorillonite features an expandable 2:1 layered structure with high swelling capacity, large specific surface area, and significant cation exchange capacity, making it particularly sensitive to the action of reagents such as salts, polymers, or nanoparticles. For instance, low concentrations of sodium or anionic polymers can induce pronounced dispersion, whereas the presence of divalent cations favors the formation of gel-like networks. Its rheological behavior is governed by “house-of-cards” structures and the thickness of the electrical double layer, which explains its tendency to exhibit high yield stresses.
(ii) Kaolinite, in contrast, possesses a non-expandable 1:1 layered structure, with a lower specific surface area and a more heterogeneous surface charge. Suspensions of kaolinite generally exhibit limited swelling and more stable viscosities over time. The response to the addition of polymers and surfactants is primarily associated with interactions at edges and structural defects, rather than interlayer adsorption; therefore, the rheological behavior of these suspensions tends to be more predictable, with reduced sensitivity to changes in pH or ionic strength.
(iii) Illite has a non-expandable 2:1 structure with low cation exchange capacity and limited surface reactivity. As a result, its response to rheology-modifying reagents is less pronounced. However, some studies have reported a slight modification of its colloidal stability using cationic surfactants and high molecular weight polymers, albeit to a lesser extent than in systems containing montmorillonite or kaolinite [152,153,154,155]. These studies suggest that, although illite exhibits lower surface reactivity, its colloidal and rheological behavior can still be altered using specific reagents, particularly under favorable chemical conditions.

## 4. Research Opportunities

In the mining industry, mineral processing involves handling large volumes of slurries containing fine particles and clay minerals. These components can adversely affect the rheological properties of suspensions, impacting key operations such as grinding, flotation, thickening, and tailings transport. Modifying the rheology of these systems is crucial for optimizing process efficiency, reducing energy consumption, and minimizing environmental impact.

Various chemical additives, including polymers, surfactants, salts, and nanoparticles, have been used to alter particle aggregation or dispersion. However, the performance of these additives under different processing conditions remains uncertain, opening new avenues for research.

This section explores several lines of investigation aimed at improving rheology modifiers. It encompasses the development of new additive formulations, their interactions with various clay minerals, the impact of environmental factors on additive efficiency, optimization for thickened tailings, and sustainable approaches utilizing eco-friendly materials. Experimental strategies are also proposed to evaluate performance and support the implementation of more efficient treatment strategies for clay-rich suspensions in the mining industry.

### 4.1. New Formulations of Rheology Modifiers

Particle interactions and the composition of the aqueous phase largely influence the rheology of clay suspensions in mining. Additives such as polymers, surfactants, and salts have been employed to improve these interactions. However, current formulations exhibit limitations such as reduced efficiency in saline environments, toxicity, or strong dependence on pH. The development of new formulations that combine different mechanisms—such as hybrid polymers and modified biopolymers—could enhance suspension stability without compromising process performance. Biopolymers offer a sustainable alternative with reduced environmental impact.

Polymers are widely used to modify the rheology of clay suspensions, with performance depending on both molecular weight and chemical structure. Low molecular weight polymers act as dispersants, while high molecular weight polymers serve as flocculants. Combining both in hybrid formulations may enhance both stability and flow, allowing better control of rheological behavior. Biopolymers derived from natural sources such as cellulose, chitosan, and alginates offer a greener alternative to synthetic polymers. However, their sensitivity to pH and salinity can limit performance in mining environments. Chemical modification of these materials may improve their robustness and compatibility.

### 4.2. Interactions Between Additives and Specific Clay Minerals

Clay minerals such as montmorillonite, kaolinite, and illite exhibit distinct structural and surface charge properties that influence their behavior in suspension. Chemical additives can modify these properties, but their effectiveness varies depending on the type of mineral and the surrounding chemical environment. Understanding how nanoparticles, surfactants, and other reagents interact with clay aggregates is essential for optimizing their use in mining processes. Research in this area will enable the design of more effective strategies to enhance either dispersion or flocculation, depending on the needs of each unit operation.

Nanoparticles have emerged as innovative agents for altering the rheology of clay-rich suspensions. Their high surface area enables efficient interaction with mineral particles, which can change aggregation patterns and improve system stability. However, the effects of different nanoparticle types on various phyllosilicates are not yet fully understood. Surfactants can significantly influence the rheology of clay suspensions by modifying interparticle forces. Depending on their charge and molecular structure, surfactants may promote dispersion or facilitate aggregate formation. These effects directly impact suspension stability and behavior in operations like flotation.

The use of multiscale modeling techniques such as Molecular Dynamics (MD) and Computational Fluid Dynamics (CFD) would be essential to complement and deepen the understanding of interactions between additives and clay minerals beyond experimental observations. MD can provide atomic-level insights into the adsorption mechanisms of polymers, surfactants, and nanoparticles onto mineral surfaces under various chemical conditions of the dispersing medium. CFD enables the simulation of the macroscopic behavior of clay suspensions during pipeline transport or handling in thickeners. The integration of experimental and simulation approaches would facilitate the optimized design of more effective additives for improved rheological performance under real mining operational conditions.

### 4.3. Impact of Physicochemical Conditions of Water on Additive Effectiveness

The rheological properties of clay-rich suspensions can vary significantly depending on temperature, pH, and salinity of the water used in processing. In particular, the increasing use of seawater (or partial seawater) in mining operations introduces higher concentrations of dissolved ions, which can affect the performance of rheological additives. Understanding how these physicochemical factors influence the behavior of modifiers will support the development of more robust reagents that can maintain performance under diverse operational conditions. Additionally, it will help define optimal parameters for controlling rheology in real industrial settings.

The properties of polymers used as rheological modifiers can be temperature sensitive. In mining environments, slurries are often subjected to significant thermal variations, depending on the location and type of operation. Evaluating the thermal stability and functionality of these polymers is essential for ensuring consistent performance. The use of seawater in mineral processing is a growing alternative for reducing freshwater demand. However, its high salinity and ionic composition can alter the effectiveness of rheological modifiers. Understanding how these ions influence dispersion and flocculation in clay systems is critical to optimizing performance under limited freshwater availability.

### 4.4. Optimization of Additives for Thickened Tailings

The management of thickened tailings is a key strategy for enhancing water recovery and minimizing the environmental impact of mineral processing. However, increasing the solids concentration in tailings results in slurries with higher viscosity and yield stress, which complicates pumping and deposition in TSFs. The integration of new additives may improve the flowability of thickened tailings without compromising their stability once deposited. In this context, the use of specialized polymers and nanoparticles could facilitate tailings transport, lower energy consumption, and prevent operational issues such as rake torque overload or bed formation in thickeners.

Due to their high viscosity and yield stress, thickened tailings pose challenges in long-distance pipeline transport. The addition of nanoparticles may reduce internal friction and enhance flow behavior, thereby decreasing the energy demand of pumping systems. The accumulation of material in high-solids thickeners can result in excessive rake torque or even complete stoppage, leading to operational inefficiencies. Enhancing the flowability of the underflow through targeted additive use may allow for more stable and continuous operation.

### 4.5. Sustainable and Eco-Friendly Approaches

The use of non-biodegradable synthetic chemical additives in mining operations can lead to environmental risks. Consequently, there is increasing interest in developing rheological modifiers derived from natural and renewable sources, such as agricultural residues and industrial by-products. The adoption of biodegradable and eco-compatible reagents could significantly reduce the environmental footprint of the mining industry.

Assessing the biodegradability and ecotoxicological impact of these new additives is critical to ensure their large-scale implementation is sustainable. Agricultural residues contain polysaccharides and other compounds with the potential to act as rheological modifiers. Utilizing these residues would reduce reliance on synthetic reagents and promote circular economy practices in the mining industry. The development of sustainable rheological control strategies must include evaluation of additive degradation, as this determines the persistence and environmental safety of such reagents in natural systems.

Biopolymers offer significant advantages in terms of biodegradability and reduced environmental impact compared to synthetic additives. However, evaluating their economic and operational feasibility under real mineral processing conditions is essential. In this context, there is a need for studies assessing the cost–benefit ratio of biopolymer uses in comparison with synthetic polymers. Both physicochemical factors, such as effective dosage and compatibility with the medium’s chemical conditions, and logistical factors, including regional availability and long-term feasibility, must be considered. Following laboratory-scale experimental validation, it will be necessary to validate these additives at pilot and industrial scales to determine their performance under real operational conditions. Ultimately, the effective implementation of these sustainable additives at the industrial level will depend on the optimal integration of economic, environmental, and technical criteria.

## 5. Conclusions

This review critically examined the current state of knowledge on chemical additives used to modify the rheological properties of clay mineral-based suspensions, with a particular focus on salts and pH modifiers, polymers, surfactants, and nanoparticles:

(i) Salts and pH modifiers influence the compression of the electrical double layer and the neutralization of surface charges, thereby altering the aggregation/disaggregation state of particles and, consequently, the rheological behavior of the suspension. However, these reagents are often treated as auxiliary variables, and their coexistence with other additives warrants more systematic investigation.
(ii) Polymers exhibit high versatility, as they can induce either dispersion or flocculation depending on characteristics such as molecular weight, molecular architecture (linear or branched), ionic nature (anionic or cationic), and applied dosage. Biopolymers are receiving increasing attention due to their biodegradability and functional diversity, although their performance under extreme salinity or temperature conditions remains insufficiently validated.
(iii) Surfactants also exert a significant influence on rheological behavior, depending on their ionic character, molecular structure, and molecular weight. Among these, ionic character is the most studied factor: cationic surfactants tend to promote flocculation via adsorption onto negatively charged clay surfaces, whereas nonionic and anionic surfactants may induce either stabilization or reversible flocculation. Nonetheless, there is a notable lack of studies evaluating surfactant behavior under industrially relevant conditions, such as seawater or complex reagent systems.
(iv) Nanoparticles possess strong potential due to their large specific surface area and tunable physicochemical properties, such as size, morphology, and surface charge, which may promote either stabilization or destabilization of clay mineral suspensions. However, their synthesis and/or surface functionalization are often associated with high costs, unexplored environmental risks, and limited compatibility with specific minerals.

This review also identified several limitations in the current literature. Many studies are conducted under idealized conditions using deionized water, which fails to capture the complexity of real process waters, such as seawater or industrial brines. There is a strong dependence on pH and mineralogical composition, and the lack of standardized experimental protocols hampers the comparability of results across studies. Furthermore, the reviewed literature rarely included pilot- or industrial-scale validation of the reagents, overlooking critical aspects such as environmental degradability and economic feasibility.

Future research should address these knowledge gaps by incorporating more realistic operational conditions, including highly saline process waters and complex system matrices. Investigations into synergistic interactions between additives—such as hybrid polymer–nanoparticle systems—are also recommended, alongside efforts to optimize multicomponent formulations. In parallel, the development of environmentally benign, biodegradable, and cost-effective additives specifically tailored for industrial applications should be prioritized. Multiscale approaches that integrate molecular modeling, laboratory-scale experiments, and pilot-scale validation should also be adopted. Ultimately, it is crucial to standardize rheological evaluation methods, enabling the correlation of measured rheological parameters with real-world performance in industrial unit operations.

## Figures and Tables

**Table 1:** Systematization of Rheology-Modifying Reagents.

Type of Additive	Common Examples	Mechanism of Action	Rheological Effect	Relevant Conditions
Salts and pH Modifiers	NaCl, CaCl_2_, MgCl_2_, NaOH, HCl	Modification of zeta potential, compression of the electrical double layer, dissociation, or protonation of functional groups	Dispersion or flocculation, depending on ion type, concentration, and pH	pH, type of salt, ionic strength, salinity, mineralogical composition
Polymers	Polyacrylamide (PAM), chitosan, CMC, carrageenan	Surface adsorption, bridging, and steric repulsion	Flocculation or stabilization, depending on molecular weight and structure	pH, salinity, temperature, dosage, presence of divalent ions
Surfactants	CTAB, SDS, DDAO, surfactin	Surface charge modification, surface tension reduction, and emulsification	Dispersion, reversible flocculation, or viscosity modification	pH, surfactant type, CMC*
Nanoparticles	Colloidal silica, iron oxides, hybrid nanoparticles	Physical/chemical interaction with surfaces, structural reinforcement, size and charge effects	Stabilization, reinforcement, or gelation of the suspension	Particle size, specific surface area, interaction with other additives

**Table 2:** Comparison of reagent advantages and limitations.

Type of Reagent	Dominant Mechanism	Main Advantages	Observed Limitations
Salts and pH	Compression of the electrical double layer, modulation of surface potential	Low cost, easy dosing, rapid response	Limited control, non-specific effect, sensitivity to ionic concentration or ion type
Polymers	Steric stabilization, interparticle bridging, charge neutralization	High efficiency, customizable formulations based on molecular weight or functional groups	Performance sensitive to salinity, pH, and mineral type; potentially high cost; variable biodegradability
Surfactants	Selective adsorption, reduction in surface tension, micelle-induced aggregation	Controlled dispersion or flocculation depending on nature (anionic, cationic, non-ionic); good performance in alkaline or saline media	Foam formation, potential toxicity, competition with other ionic species, and sensitivity to CMC*
Nanoparticles	Surface adsorption, heteroflocculation, electrostatic or magnetic interaction	High specific surface area, effective at low doses, good tolerance to adverse conditions	Synthesis or functionalization cost, environmental risks still underexplored, and selective mineral compatibility

## Data Availability

The original contributions presented in this study are included in the article. Further inquiries can be directed to the corresponding authors.
